# What explains the regional variation in the use of general practitioners in Australia?

**DOI:** 10.1186/s12913-020-05137-1

**Published:** 2020-04-19

**Authors:** Chunzhou Mu, Jane Hall

**Affiliations:** 1 Business School, Jilin University, Changchun, 130012 China; 2 Centre for Health Economics Research and Evaluation (CHERE), University of Technology Sydney, Level 2 Building 5 Block D, 1-59 Quay St., Haymarket, NSW 2000 Australia

**Keywords:** GP usage, Regional variation, Statistical local areas, Australia

## Abstract

**Background:**

Regional variation in the use of health care services is widespread. Identifying and understanding the sources of variation and how much variation is unexplained can inform policy interventions to improve the efficiency and equity of health care delivery.

**Methods:**

We examined the regional variation in the use of general practitioners (GPs) using data from the Social Health Atlas of Australia by Statistical Local Area (SLAs). 756 SLAs were included in the analysis. The outcome variable of GP visits per capita by SLAs was regressed on a series of demand-side factors measuring population health status and demographic characteristics and supply-side factors measuring access to physicians. Each group of variables was entered into the model sequentially to assess their explanatory share on regional differences in GP usage.

**Results:**

Both demand-side and supply-side factors were found to influence the frequency of GP visits. Specifically, areas in urban regions, areas with a higher percentage of the population who are obese, who have profound or severe disability, and who hold concession cards, and areas with a smaller percentage of the population who reported difficulty in accessing services have higher GP usage. The availability of more GPs led to higher use of GP services while the supply of more specialists reduced use. 30.56% of the variation was explained by medical need. Together, both need-related and supply-side variables accounted for 32.24% of the regional differences as measured by the standard deviation of adjusted GP-consultation rate.

**Conclusions:**

There was substantial variation in GP use across Australian regions with only a small proportion of them being explained by population health needs, indicating a high level of unexplained clinical variation. Supply factors did not add a lot to the explanatory power. There was a lot of variation that was not attributable to the factors we could observe. This could be due to more subtle aspects of population need or preferences and therefore warranted. However, it could be due to practice patterns or other aspects of supply and be unexplained. Future work should try to explain the remaining unexplained variation.

## Background

Regional variation in the use of health care services is widespread and persistent over time [1–3]. Some of this variation reflects differences in population needs and/or preferences, and can be considered warranted; however, factors on the supply side, such as variations in medical practice styles and differential access to health care services should be considered unexplained in a health system which aims to provide equal access. This may suggest that people in low-use areas are underserved, or that in high-use areas services delivered are of marginal benefit, which results in inefficiency and inequity in the health care system. Therefore, identifying and understanding what drives regional variation can contribute to improving health system performance.

There is a vast literature on regional variation in health care use. However, the majority of papers focus on hospital admissions or surgical procedures, such as hospitalisation rates, utilisation of knee replacements, and hip fracture repair, etc. [4–7]. Regional variation in primary care utilisation has received relatively little attention, despite the key role of primary care in most developed country health systems. Past studies suggest that regional variations in the utilisation of primary care physicians’ service are driven by both demand-side factors, such as patient preferences, health status, and income, as well as supply-side factors such as provider financial incentives or practice norms [8–10]. For example, M Bech and J Lauridsen [8] studied the determinants of general practitioners (GPs) expenditure per capita across Danish municipalities. Including adjustments for spatial spillover and regional fixed effects, they found that GP density, the proportion of people aged 80 or above, and the proportion of people residing in urban areas had a statistically significant and positive effect on public GP expenditure per capita. PA Camenzind [9] used Swiss data to analyse the factors that influence regional variations in the utilisation of GP services. He found that a larger population, higher densities of GPs, higher income levels, a smaller hospital bed density, and a lower unemployment rate lead to higher use of GP services.

Australian evidence on regional variation in the use of GPs is very limited. One study using Australian data at Statistical Local Area (SLA) level to investigate the effect of socio-economic status and geographic remoteness on GP utilisation, G Turrell, BF Oldenburg, E Harris and D Jolley [11] found that the relationship between socio-economic disadvantage and GP use varied by geographic remoteness. Specifically, in metropolitan areas that had relatively unrestricted access to goods and services, more socio-economic disadvantage gave rise to higher use of GPs; however, among remote/very remote areas with restricted accessibility of services, the most socio-economically disadvantaged areas had the lowest GP use, suggesting a positive correlation. This study, though, used 1996/97 data and it is not clear that these patterns would hold some 20 years later.

We revisited this topic in the context that the distribution of GPs is unbalanced across areas and does not correspond with need in Australia in recent years [12–16]. There is higher availability of GPs in urban and inner regional areas [17], although there is also variation within urban areas (as shown in our data). There are also numbers of Australians reporting delaying or not visiting a GP when needed [18]. This suggests unequal access to health care, and it may contribute to disparities in health outcomes across areas in the long run.

We investigated regional variation using data from the Social Health Atlas of Australia by SLA which provides data on a range of demographic, social and health factors [19]. We aim to examine what factors drive regional variation and how much variation can be attributed to factors from the demand side and factors from the supply side.

### Institutional background

Medicare is Australia’s publicly funded universal health care system, providing free care in public hospitals and subsidised medical services and pharmaceuticals to all residents of Australia. In addition to Medicare, the Department of Veterans’ Affairs (DVA) is responsible for providing a variety of services to assist veteran and defence force communities, including delivering health care and rehabilitation services and providing compensation and income support entitlements. Australians are entitled to receive a fixed rebate to cover the cost of their physicians’ consultations from Medicare, as set out in the Medicare Benefits Schedule (MBS). However, physicians determine their own fees and may charge patients a fee beyond what is reimbursed by Medicare. Under Medicare, GPs are the main providers of primary care and act as gatekeepers for specialised services, constituting the first point of contact between the medical profession and the public. In addition to providing direct treatment, GPs also refer patients who need more specialised care to other medical practitioners. Each year more than 80% of Australians visit a GP [20]. Most GP services are provided without charge to the patient, and financial incentives encourage this for vulnerable groups.^1^ Recent reforms have aimed to strengthen primary care, improve access to care and reduce gaps in available services. Yet widespread variation in the use of GP services is evident. In 2011–12, GP visits per capita ranged from 7.4 to 2.4 across regional areas in Australia, being three times higher in some areas than others [21].

Emergency care is a critical and an expensive component of the health system. Presentations to the emergency department (ED) are free to patients in all public hospitals in Australia. In addition to seeking acute unscheduled care, people sometimes attend EDs for reasons that could be addressed by non-hospital services such as GPs, thus suggesting a substitution between the use of ED and GP services especially for non-urgent scenarios. The Patient Experience Survey conducted by Australian Bureau of Statistics in 2017–18, showed among those who visited an ED in the last 12 months, 18% thought that care could have been provided by a GP instead [22]. Additional evidence supports the view that strengthening primary care access and services might reduce the use of ED [23, 24].

Rurality of patients’ location of usual residence has also been shown to influence health service use in the following two ways. On the one hand, people in rural and remote areas have poorer health status than their metropolitan counterparts. They generally score worse on a range of health status measures, for example, having higher mortality rates, a higher number of chronic conditions, and higher levels of mental health concerns [25]. Also, people in regional areas are more likely to smoke, be overweight, and to engage in risky alcohol consumption. On the other hand, rural populations generally have poorer access to health services, due to an inadequate supply of hospital and other health services and workforce shortages in these areas.

## Methods

### Data source and sample construction

Data were drawn from the Social Health Atlas (SHA) of Australia by SLA, which was released by the Public Health Information Development Unit (PHIDU).^2^ The SHA brings together a range of data on population health, health service use and the social determinants of health. SLAs are the principal regional building blocks defined by the Australian Bureau of Statistics (ABS) and, in aggregate, cover the whole of Australia without gaps or overlaps. In total, there are 1397 SLAs [26],^3^ although only 1094 SLAs are included in the SHA data.^4^ Although SHA data were released in 2010, 2011, 2012, 2013, and 2014 respectively, some variables have not been updated over years. For example, for the number of GP visits, the variable of main interest, only 1 year’s observation (2009–10) has been reported. The final sample size is 756 after dropping observations with missing values.^5^ Comparing with the original sample, the SLAs in our analysis sample seem to be more socio-economically advantaged and with a higher proportion in urban areas (Additional file 1). SLAs dropped from the original sample are mainly those located in remote areas; given the geography and population distribution of Australia, the provision of health care faces challenges that are substantially different to those in larger population centres and are addressed through different policies. Therefore, our results provide estimates on the effect of demand and supply factors on regional variations in GP use primarily for non-remote Australia.

### Dependent variable

The use of GPs was measured by the number of GP visits per capita in 2009–10 by SLAs. This was calculated by dividing the total number of GP services in SLAs, including those within the MBS and DVA, by the population size in each SLA.

### Independent variables

In general, variation in regional health care utilisation is related to differences in populations’ needs for health care and in supply factors that include accessibility of services, practice patterns of health care providers, and health care system characteristics.

Need-related factors include the wide-ranging determinants of population health, burden of disease, demographics, and socioeconomic status. These factors reflect justified causes of variation in healthcare utilisation. Demographics were captured by the proportion of each age subgroup (age 0–9, 10–29, 30–44, 45–64, and 65 and above) in 2009, the percentage of males within the population in SLAs in 2009, and the percentage of the Aboriginal and Torres Strait Islander population in 2006.

We measured the health status of each local population using four indicators. First is the proportion of people who reported fair or poor health in each SLA. Second is the proportion of people with profound or severe disability living in the community. Third is the share of people aged 18 years and over with high or very high level of psychological distress. The last group of variables describes chronic diseases and conditions, which were measured by the proportions of people with Type 2 diabetes, circulatory system disease, and respiratory system disease, respectively. In this study, we used health status variables from previous years (2007–08) rather than those reported in 2009–10, to minimise the risk of bias due to reverse causality, because health status variables from the same year as GP usage (2009–10) may measure population health status after receiving care or treatment by GPs.

Health-related behaviours or health indicator variables were taken into account by the four variables: (1) percentage of current smokers among those aged 18 years and over, (2) percentage of people consuming alcohol at levels considered to be a high risk to death among those aged 18 years and over, (3) percentage of people who are physically inactive among those aged 15 years and over, and (4) percentage of obese people among those aged 18 years and over. The four variables were calculated by dividing the number of people who had these health-related behaviours in 2007–08 by the population size of each SLA. Concession cards provide access to cheaper medicines and concessions on health services in Australia, therefore the concession card status of the population was also controlled for.

The Socio-Economic Indexes for Areas (SEIFA) - Index of Relative Socioeconomic Disadvantage (IRSD) reported in 2006 was utilised to measure the socio-economic characteristics of each SLA. The IRSD identifies and ranks areas in terms of their relative socio-economic disadvantage. A low index score on the IRSD indicates relatively greater disadvantage in general, while a high score on it corresponds to a relative lack of disadvantage.^6^ To account for the non-linear effect of the IRSD, we introduced a four-category variable, where the lowest quartile consists of areas with the lowest IRSD scores (most disadvantaged).

Rurality of people’s location of residence has been shown to play a role in population health status and their accessibility to health care services. The measure of remoteness was obtained from matching the SLAs with the remoteness areas defined by the Australian Standard Geographical Classification (ASGC) remoteness index in 2011 [27]. The ASGC remoteness index provided by the ABS comprises major cities, inner regional, outer regional, remote, and very remote areas [28]. Due to the small number of SLAs in the last two categories of the ASGC, we combined the last three groups and adopted a three-level measure: (1) major cities, (2) inner regional areas, (3) rural and remote areas, including outer regional, remote, and very remote areas.

A series of measures of people’s access to health care in SHA data was also taken into consideration. They were: The proportion of people aged 18 and over who delayed purchasing prescribed medication because they could not afford it and the proportion of people who often has a difficulty with transport or cannot get to places needed. Also included is a general measure of service availability where services include banking, legal, employment and other government services as well as health care; this is the proportion of people who reported difficulty in accessing services in 2007–08.

Supply-related factors, generally relating to unjustified variation, were also included in the analysis. In this study, we used the density of GPs and specialists to capture the capacity of the health care system and the accessibility of health care. GP and specialist densities by local government areas (LGAs) were constructed from the Health Workforce Data provided by Australian Institute of Health and Welfare (AIHW). The two variables were measured by the number of GPs and specialists per 1000 population at LGA level in 2010 separately, based on the correspondence between LGA and SLA [29, 30]. Each LGA is formed by one or more SLAs and there was a total of 667 LGAs in Australia in 2011. Additionally, to account for the substitutability between ED treatment and GP usage, the number of EDs in each SLA was constructed and included in the analysis.^7^

The variable names and definitions used in this paper and the mean and standard deviations of these variables are summarised in Table 1. The number of GP attendances per person by SLAs ranged from 2.35 to 9.27, with an overall average of 5.58, indicating substantial regional variation in GP use in Australia.

**Table 1:** Definitions of variables and descriptive statistics

Variable name	Definition	Mean	SD
*Dependent variables*			
Number of GP visits per capita	=Total GP services (MBS and DVA) in SLAs/population in each SLA in 2009–10	5.58	1.08
*Explanatory variables: demand-side factors*			
Age distribution			
Age 0–9	Proportion of population aged 0–9	0.12	0.02
Age 10–29	Proportion of population aged 10–29	0.26	0.05
Age 30–44 (base group)	Proportion of population aged 30–44	0.20	0.03
Age 45–64	Proportion of population aged 45–64	0.27	0.04
Age 65 and above	Proportion of population aged 65 and over	0.14	0.05
Share of male	Proportion of male population	50.33	1.86
ASGC remoteness index			
Major city (base group)	=1 if in major city	0.46	0.50
Inner regional area	=1 if in inner regional areas	0.26	0.44
Rural and remote areas	=1 if in outer regional, remote, and very remote areas	0.28	0.45
SEIFA-IRSD index			
25th percentile and below (the most disadvantaged)	=1 if below 25th percentile of SEIFA	0.20	0.40
25th–50th percentile	=1 if 25th–50th percentile and below of SEIFA	0.27	0.44
50th–75th percentile	=1 if 50th–75th percentile and below of SEIFA	0.25	0.44
Above 75th percentile (base group - the most advantaged)	=1 if above 75th percentile of SEIFA	0.28	0.45
Proportion of Aboriginal population	Proportion taken up by Aboriginal population	3.53	1.08
Proportion of concession card holders	Proportion of population holding concession cards	10.98	1.92
Share of fair or poor self-assessed health population	Share of people who report fair or poor self-reported health in each SLA	14.67	3.75
Chronic disease and conditions (%)			
Type 2 diabetes	Proportion of population having Type 2 diabetes	3.53	0.83
Circulatory system disease	Proportion of population having circulatory system disease	22.71	5.34
Respiratory system disease	Proportion of population having respiratory system disease	26.10	2.96
Proportion of people with profound or severe disability living in the community	Proportion of people who have profound or severe disability living in the community	3.53	1.08
Proportion of people with high/very high level of psychological distress	Proportion of people who have high/very high level of psychological distress	10.98	1.92
Health-related factors (%)			
Current smokers	Proportion of current smokers (aged 18 and above)	20.35	3.75
Alcohol consumption at levels of high risk to health	Proportion of people consuming alcohol at levels of a high risk to health (aged 18 and above)	5.83	2.26
Physical inactivity	Proportion of persons who are physically inactive (aged 18 and above)	35.99	6.05
Obese persons	Proportion of persons who are obese (aged 18 and above)	18.03	3.23
Access to services (%)			
Delayed purchasing prescribed medication	Proportion of people aged 18 years and over who delayed purchasing prescribed medication because they could not afford it	8.67	2.75
Have difficulty in accessing service	Proportion of people aged 18 years and over who had difficulty in accessing services	25.03	4.54
Have difficulty in transportation	Proportion of people aged 18 years and over who often has a difficulty with transport or cannot get to places needed	3.03	0.87
*Explanatory variables: supply-side factors*			
Physician density			
Number of specialists per 1000 population	Number of GPs per 1000 population in each LGA	0.85	2.59
Number of GPs per 1000 population	Number of specialists per 1000 population in each LGA	1.09	0.50
Number of EDs by SLAs			
No ED (base group)	=1 if there is no EDs in a SLA	0.38	0.49
1–2 EDs	=1 if there are 1–2 EDs in a SLA	0.44	0.50
3 or more EDs	=1 if there are 3 or more EDs in a SLA	0.18	0.38

### Empirical strategy

The analysis was undertaken in two stages. The first aim of this study is to examine the factors that influence regional variation in GP use and to ascertain the relative impact of various control variables on the magnitude of the differences in GP usage. To begin, the ordinary least squares (OLS) regression model was estimated with the number of GP visits per capita by SLAs as the outcome variable. Following this, to further explore the variability in effects of the explanatory variables across the distribution of GP use, quantile regression (QR) models were also utilised. Since there are noticeable differences in the provision of primary health care between rural and remote areas and major cities in terms of GPs’ services hours, travelling distances for GPs, and models of medical care [31], we also performed a subsample analysis by rurality to allow the effect of factors that influence the use of GP services to vary between urban and rural areas.

In the second stage of the analysis, we targeted unexplained differences in the use of GP consultations between two extreme groups — areas in the top and bottom quintiles of the distribution of the GP usage. It is the comparison between these two groups that is more challenging and makes us think about whether there is under or over use. We estimated a series of multiple linear regression models that initially include only categorical indicators representing areas’ quintiles rankings of GP usage. The coefficients of the quintile dummy variables measure the difference in GP visits per capita between quintile 1 (with the lowest number of visits) and each of the other four quintiles. With no other control variables in the model, these initial coefficients are precisely the differences in the number of GP visits per capita across the quintiles. We then expanded the number of explanatory variables in the model with demand-side and supply-side variables entering into the model sequentially. The coefficients of the quintile dummy variables change as each set of additional measures were included; therefore, the changes in these coefficients represent the amount of the initial regional difference that can be “explained” by the additional measures. The coefficients of the quintile dummy variables from the final regression model that includes all the observable independent variables represent the amount of the difference that is due to unidentified factors.

## Results

To examine how much of the regional variation in GP visits per capita can be statistically explained by certain factors, we first estimated the OLS model with only the control variables of health care need factors. After this, we added supply-side factors to the regression. This strategy has been commonly utilised in analysing regional variation in healthcare spending [1, 32]. In addition to adopt R-squared value to quantify how well the model fits the data, we also used standard deviation of the residuals, which is determined by the standard deviation of all residuals after regression in each step, following a previous study by D Göpffarth, T Kopetsch and H Schmitz [32]. The more we can explain by observable control variables, the lower the standard deviation becomes. Table 2 shows the explanatory share of various control variables. It can be seen that medical need, that is, differences in health status and demographic structure, explains a small proportion of regional variations in the number of GP visits per capita. Specifically, a regression with only control variables of demand-side factors had an R-squared of 51.78% and reduced the standard deviation of GP usage by 30.56%. Supply-side factors increased the explanatory share to 32.24%. Therefore, when all the explanatory variables were taken into account, we can reduce the standard deviation of GP-consultations per capita by 32.24%.^8^

**Table 2:** Reduction in variation

Model	R-squared (%)	Standard deviation of residuals	
		Std. dev.	Reduction (%)
Unadjusted model	0	1.075	0
Model with control variables			
Demand-side variables	51.78	0.747	30.562
Demand-side + supply-side variables	54.04	0.729	32.240

*Notes*: The complete regression results for the specification with all observable control variables are to be found in Table 3

Table 3 presents the individual parameters of the estimates obtained from estimating an OLS model and QR for the 25th, 50th, and 75th percentile. Robust standard errors for OLS and bootstrapped standard errors for QR were reported to obtain heteroscedasticity-robust estimates.^9^ On the demand side, there was evidence that a higher regional GP consultation rate was found to be associated with a higher percentage of population who hold concession cards, who have profound or severe disability, and who are obese across all quantiles. The coefficients of the variable indicating the presence of difficulty in accessing services were negative and statistically significant throughout all quantiles. The proportion of population with Type 2 diabetes negatively affected regional GP use. A positive correlation existed between the share of elderly people and the frequency of GP visits, as indicated by the OLS results. Additionally, the results for OLS regression also suggested that areas located outside major cities have lower GP use than those in major cities and the gap in the frequency of GP-consultations widened with increasing remoteness. We can see that the effects of regional remoteness on GP use varied with the level of GP use. At the lower quantile, the pattern of the results was similar to those obtained using OLS. At a higher quantile, the impact of remoteness became statistically insignificant, suggesting that the regional remoteness had an effect only at the lower end of the distribution of GP use. The rest of the control variables related to need for health care, such as the gender distribution of the local population, the proportion of Aboriginal population, and the share of population with fair or poor health status, the share of people with high level of psychological distress, and the social-economic status of local areas did not significantly affect the use of GP services.

**Table 3:** Estimation results for regional variation in GP use

Variables	Dependent variable: Number of GP visits per capita by SLAs							
	OLS		Quantile 0.25		Quantile 0.5		Quantile 0.75	
	(1)		(2)		(3)		(4)	
Age distribution (base is age 30–44, %)								
Age 0–9	0.001	(0.029)	0.004	(0.039)	− 0.008	(0.035)	−0.021	(0.036)
Age 10–29	− 0.002	(0.015)	− 0.032	(0.021)	− 0.012	(0.021)	− 0.010	(0.020)
Age 45–64	− 0.009	(0.016)	−0.018	(0.018)	0.006	(0.023)	0.000	(0.021)
Age 65 and above	0.071**	(0.031)	0.029	(0.033)	0.072*	(0.040)	0.076*	(0.044)
Share of male	0.023	(0.027)	−0.022	(0.037)	0.013	(0.035)	0.010	(0.030)
ASGC remoteness index (base is major city)								
Inner regional areas	−0.505***	(0.164)	−0.735***	(0.203)	−0.503***	(0.186)	−0.109	(0.239)
Rural and remote areas	−0.610***	(0.231)	−0.944***	(0.269)	−0.534*	(0.273)	−0.012	(0.349)
SEIFA-IRSD index (base is above 75th percentile - the most advantaged)								
25th percentile and below (the most disadvantaged)	−0.119	(0.202)	−0.061	(0.246)	0.054	(0.232)	0.052	(0.298)
25th–50th percentile	−0.139	(0.157)	−0.203	(0.167)	−0.023	(0.182)	0.095	(0.235)
50th–75th percentile	0.063	(0.103)	0.059	(0.121)	0.155	(0.103)	0.263*	(0.149)
Proportion of Aboriginal population	0.007	(0.013)	−0.015	(0.019)	0.003	(0.020)	0.026	(0.019)
Proportion of concession card holders	0.059***	(0.012)	0.065***	(0.017)	0.047**	(0.018)	0.067***	(0.019)
Share of fair or poor self-assessed health population	−0.026	(0.020)	− 0.010	(0.023)	− 0.022	(0.031)	− 0.059*	(0.036)
Chronic disease and conditions (%)								
Type 2 diabetes	−0.576***	(0.158)	−0.509***	(0.179)	−0.574***	(0.219)	−0.442*	(0.239)
Respiratory system disease	−0.011	(0.013)	−0.028*	(0.017)	−0.023	(0.017)	−0.011	(0.018)
Circulatory system disease	0.003	(0.021)	0.019	(0.027)	−0.010	(0.025)	−0.048	(0.029)
Proportion of people with profound or severe disability living in the community	0.318***	(0.083)	0.218**	(0.094)	0.305***	(0.105)	0.287**	(0.115)
Proportion of people with high/very high level of psychological distress	0.108*	(0.055)	0.075	(0.061)	0.105	(0.072)	0.093	(0.078)
Health-related factors (%)								
Current smokers	−0.054**	(0.026)	−0.032	(0.032)	−0.045	(0.030)	−0.058	(0.040)
Alcohol consumption at levels of high risk to health	−0.015	(0.021)	0.020	(0.031)	−0.006	(0.027)	−0.052*	(0.031)
Physical inactivity	0.010	(0.012)	0.020	(0.015)	0.016	(0.016)	0.008	(0.016)
Obese persons	0.099***	(0.027)	0.041	(0.035)	0.066**	(0.033)	0.134***	(0.037)
Access to services (%)								
Delayed purchasing prescribed medication because could not afford it	−0.024	(0.025)	−0.019	(0.029)	0.007	(0.028)	−0.028	(0.032)
Have difficulty in accessing service	−0.060***	(0.018)	−0.050**	(0.022)	−0.072***	(0.021)	−0.087***	(0.027)
Have difficulty in transportation	−0.142*	(0.076)	−0.124	(0.093)	−0.121	(0.091)	−0.080	(0.104)
Physician density								
Number of specialists per 1000 population	−0.057***	(0.013)	−0.051**	(0.024)	−0.066***	(0.021)	−0.072***	(0.025)
Number of GPs per 1000 population	0.164*	(0.087)	0.201**	(0.097)	0.246**	(0.105)	0.333**	(0.154)
Number of EDs by SLAs (base is no EDs)								
1–2 EDs	0.100	(0.071)	0.112	(0.081)	0.121	(0.088)	0.153*	(0.090)
3 or more EDs	0.080	(0.117)	−0.034	(0.144)	0.070	(0.175)	0.045	(0.172)
Constant	3.866**	(1.916)	7.273**	(2.893)	5.071**	(2.567)	6.001***	(2.164)
Number of observations	756		756		756		756	
R squared (Pseudo R squared)	0.540		0.364		0.333		0.343	

*Notes:* Numbers in parentheses are white robust standard errors. Significant level * *p* < 0.10, ** *p* < 0.05, *** *p* < 0.01

Turning to the supply-side variables, the density of GPs and specialists, as a measure of the accessibility of service, was found to be correlated with the variations in GP use. Specifically, the availability of more GPs in the local areas resulted in higher use of GP services while the supply of more specialists reduced it. These effects were statistically significant throughout the quantiles. However, there was no evidence that population’s use of ED and GP services were substitutes for each other; all the estimated coefficients for ED variables were statistically insignificant. A visual comparison of the estimates across the whole distribution (i.e. 10 quantiles) of GP usage was displayed (see Additional file 2).

There was heterogeneity in the effects of the factors that influence the GP usage in urban and rural and remote areas (see Additional file 3). In terms of age distribution differentials, a higher proportion of population aged 0–9 and aged 10–29 led to fewer GP visits and a higher proportion of population aged 65 and over triggered higher GP utilisation, relative to the middle age group. However, these effects were only statistically significant among areas located in urban regions. In terms of socio-economic status, the more disadvantaged areas had fewer GP visits than the less disadvantaged areas, with the magnitude being larger for the rural and remote areas. Local population’s health status, health-related behaviours, the presence of difficulty in accessing services, and the density of GPs and specialists only affected GP usage for urban areas but not for rural and remote areas.

**Fig. 1:**
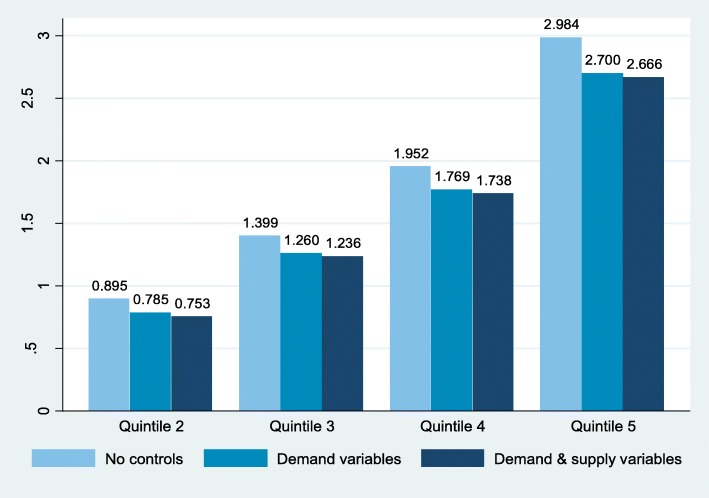
Unexplained differences in GP visits between the quintile with lowest GP usage and higher-usage quintiles. *Notes:* The unexplained differences in GP visits per capita were expressed as coefficients of quintile dummy variables. The division into five quintiles was based on unadjusted GP visits per capita. The three models were a series of linear multiple linear regression models that used GP visits per capita as the dependent variable and differed in the sets of covariates added as independent controls. The first model contained only dummy variables for the quintiles, representing the differences in GP usage across the quintiles. The second model added the variables from demand-side, such as age, gender, and health-related indicators. The third model contained supply-side factors as well as demand-side variables: the density of GP and specialists and the number of EDs. All the estimated coefficients were statistically significant

A comparison of GP usage in the top quintile with the respective figures for those in the lowest quintile was taken as an alternative measure of the variation in this study. All the SLAs are ranked according to their GP usage and divided into quintiles. The unadjusted GP visits per capita was 72.13% higher in geographic regions in the highest usage quintile than in regions in the lowest quintile, with the mean GP visits per capita ranging from 4.137 in quintile 1 to 7.121 in quintile 5, a difference of 2.984. The estimated coefficients of categorical indicators for quintiles rankings of GP usage in a series of models measure the difference in regional GP use between the lowest quantile (the base group) and each of the other four quintiles. The model is built step wise with factors from demand-side and those from supply-side entering into the model sequentially.^10^ Fig. 1 visualises the results and compares how far the quintiles were from each other after each step of the adjustment. We can see that, after adjustment for demand- and supply-side factors successively, the magnitude of the unexplained difference in the use of GP services between the highest and lowest quintiles dropped from 2.984 to 2.700, and to 2.666, or from 72.13 to 65.26%, and to a final 64.44%, suggesting that the observed geographic differences could be explained in part by differences in patients’ need and the supply of physician workforce. However, in our analyses, the percentage of GP usage differences between top and bottom quintiles that remained unexplained was still substantial, at over 60%. Figure 1 also highlights that there was hardly any change to the sequence of the ranking for GP utilisation. The rankings of the quintiles retained through all adjustment steps, despite the fact that SLAs were divided up into groups on a basis of their unadjusted GP usage. The same exercise was also performed after we divided all the SLAs into deciles based on the rankings of GP usage and robust results were found (Additional file 4).

## Discussion

Higher per capita GP-consultation rate was found to be consistently influenced by population characteristics such as age (older) and health status (worse). With respect to age, our findings are consistent with the findings in EF de Vries, R Heijink, JN Struijs and CA Baan [1] and A Busato and B Künzi [33]. The results also revealed that higher GP usage was associated with poor health status of the local population, i.e. a higher percentage of population who are obese or who have profound or severe disability. However, the results for the presence of certain chronic conditions differed from those of S Zuckerman, T Waidmann, R Berenson and J Hadley [34] whose results based on US data suggested that having heart disease and nonskin cancer led to higher health care utilisation. In this study, respiratory disease and circulatory system disease did not significantly affect the use of GP services. Moreover, the proportion of population with Type 2 diabetes was found to negatively affect the frequency of GP use, which was unexpected. We expect that more diabetics would indicate a greater need for more medical services and therefore more GP-consultations. One possible explanation is that individuals with established diabetes are more likely to consult specialists. However, we do not observe the use of GP services by individuals with diabetes, so we are unable to identify whether this is lower use by diabetic patients, or lower use in general. Another plausible explanation is that this patient group is less adherent to treatment recommendations and therefore, less likely to seek care from professionals. In terms of the supply-side factors, the availability of more GPs in the local areas was found to lead to higher rates of the utilisation of GP services while the supply of more specialists reduced it; findings accord with results from Australia and Switzerland [11, 33].

Our study has some limitations. The data available were aggregate level so we were unable to investigate patterns of use by sub-groups, such as diabetic patients in high vs. low use areas. Similarly, we were unable to analyse characteristics of health care markets by region, including physician practice patterns. Finally, these were cross-sectional and non-experimental data and the results cannot be interpreted as evidence of causation.

However, the findings raise issues of equity and efficiency. Our results demonstrate substantial variation in GP utilisation across Australian regions with only a small proportion explained by population health needs. In a health system which aims to provide equal treatment for equal need this high level of clinical variation is not warranted. This unexplained variation is only partially explained by the supply of GPs. Moreover, in general there is no evidence that higher use of resources leads to better outcomes than in areas where less intervention is practiced [35]. Interestingly the number of specialists was found to be negatively correlated with GP use in this study, suggesting that rather than low GP use indicating inequity of access, rather it reflects good access to specialists. Therefore, high GP use may indicate poorer access to care. This requires further research to establish the extent to which specialist care and GP care deliver similar health outcomes, and under what circumstances.

While our results supported the substitutability of GP and specialist visits, they do not show the same relationship between GP and ED visits, although a shortage of GPs is generally assumed to increase ED visits. Further research is needed to improve our understanding of the drivers of health service utilisation, and the patterns of substitution across services to improve health system performance.

## Conclusion

This study examined the factors that contribute to regional variation in GP use: both factors determining populations’ demand for health care and supply-side factors were found to influence regional differences in the number of GP visits. The demand-side factors explain 30.56% of the variation as measured by the standard deviation of adjusted GP-consultation rate and controlling for supply-side factors additionally increased the explanatory share to 32.24%. The major proportion of variation remains unexplained by the factors we could observe.

## Supplementary information

**Additional file 1: Table S1.** Comparison of the characteristics of original sample and sample used in the analysis (restricted sample).

**Additional file 2: Figure S1.** Coefficients of variables across quantiles.

**Additional file 3: Table S2.** Estimation results for regional variation in GP use by rurality.

**Additional file 4: Figure S2.** Unexplained differences in GP visits between the decile with lowest GP usage and higher-usage deciles.

## Data Availability

The datasets generated and/or analysed during the current study are drawn from Social Health Atlas of Australia by Statistical Local Area (2010–2014), which is released by Public Health Information Development Unit (PHIDU), http://phidu.torrens.edu.au/social-health-atlases/data-archive/data-archive-social-health-atlases-of-australia.

## Abbreviations

GPs: General practitioners
DVA: Department of Veterans’ Affairs
MBS: Medicare Benefits Schedule
ED: Emergency department
SLA: Statistical Local Area
ABS: Australian Bureau of Statistics
SHA: Social Health Atlas
PHIDU: Public Health Information Development Unit
SEIFA: Socio-Economic Indexes for Areas
IRSD: Index of Relative Socioeconomic Disadvantage
ASGC: Australian Standard Geographical Classification
LGA: Local government areas
AIHW: Australian Institute of Health and Welfare
QR: Quantile regression
OLS: Ordinary least squares

## Footnotes

1 For example, vulnerable groups include people who are less than 16 years old, or those who are concession card holders. Concession card holders are predominantly aged pensioners, certain social security allowance recipients, and people from low-income families. They are entitled to access to prescription medicines at a cheaper rate. Also, patients with concession cards are more likely to be bulk billed or charged lower fees by physicians than the general patients.

2 These data are part of the Public Health Information Development Unit’s Social Health Atlas series

3 The delimitation of SLAs is based on the boundaries of incorporated bodies of local government. These bodies are the Local Government Councils and the geographical areas which they administer

4 The statistics for Australian Capital Territory are missing and there is no data for the areas that are unincorporated in the corresponding state or with unknown ABS cell adjustment

5 The 756 SLAs account for around 70% of all 1094 SLAs contained in the raw data set while around 87% of the whole population have been covered by them, indicating that SLAs with lower population were dropped.

6 The score for Australia is 1000 as a benchmark

7 The information on EDs, such as name, hospital type (public or private), postcode, and address are obtained from MyHospitals, accessed at <http://www.myhospitals.gov.au/>

8 The explanatory power of various control variables also depends on the sequences in which these variables enter the regression. We consider that controlling for variables measuring medical need is the natural sequence to start with. Permuting the order of the control variables, i.e. controlling for supply-side factors first and then adding demand-side factors into the regression, changed the results slightly: supply-side factors reduced the standard deviation of GP usage by about 2% and demand-side factors increased the explanatory share to 32.24%.

9 Given that the densities of GPs and specialists are constructed at the LGA level, we undertake an analysis with standard errors being clustered at LGA level. Robust results are obtained and are available upon request

10 Full sets of results are available upon request
