# Supplementary material for: What explains the regional variation in the use of general practitioners in Australia?
Source: BMC Health Serv Res. 2020 Apr 19;20:325. doi: 10.1186/s12913-020-05137-1 (PMC7168818; doi:10.1186/s12913-020-05137-1)
Supplement: Supplementary file 1 — Additional file 1: Table S1. Comparison of the characteristics of original sample and sample used in the analysis (restricted sample). [file 12913_2020_5137_MOESM1_ESM.docx]

Additional file 1

Table A1: Comparison of the characteristics of original sample and sample used in the analysis (restricted sample)

|  | Original sample (N=1,094) | | Restricted sample (N=756) | |
| --- | --- | --- | --- | --- |
| Variables | Mean | Std. Dev | Mean | Std. Dev |
| GP visits per capita | 5.220 | (1.389) | 5.581 | (1.075) |
| Age distribution (%) |  |  |  |  |
| Age 0-9 | 13.343 | (3.728) | 12.466 | (2.443) |
| Age 10-29 | 26.810 | (5.782) | 26.390 | (5.194) |
| Age 30-44 | 20.496 | (3.492) | 20.329 | (3.285) |
| Age 45-64 | 26.105 | (4.851) | 26.516 | (3.992) |
| Age 65 and above | 13.246 | (5.237) | 14.300 | (4.663) |
| Share of male (%) | 50.915 | (2.832) | 50.328 | (1.857) |
| ASGC remoteness index |  |  |  |  |
| Major city | 0.352 | (0.478) | 0.458 | (0.499) |
| Inner regional areas | 0.247 | (0.431) | 0.258 | (0.438) |
| Rural and remote areas | 0.402 | (0.490) | 0.284 | (0.451) |
| SEIFA-IRSD index |  |  |  |  |
| 25th percentile and below (the most disadvantaged) | 0.250 | (0.433) | 0.198 | (0.399) |
| 25th-50th percentile | 0.250 | (0.433) | 0.266 | (0.442) |
| 50th-75th percentile | 0.250 | (0.433) | 0.254 | (0.436) |
| 75th percentile and above (the most advantaged) | 0.249 | (0.433) | 0.282 | (0.450) |
| Proportion of Aboriginal population | 8.118 | (19.590) | 2.926 | (4.889) |
| Proportion of concession card holders | 24.399 | (8.925) | 24.193 | (7.720) |
| Share of fair or poor self-assessed health population | 14.779 | (3.819) | 14.672 | (3.753) |
| Chronic disease and conditions (%) |  |  |  |  |
| Type 2 diabetes | 3.524 | (0.851) | 3.533 | (0.829) |
| Circulatory system disease | 16.613 | (3.984) | 16.815 | (4.024) |
| Respiratory system disease | 25.830 | (2.870) | 26.103 | (2.958) |
| Proportion of people with profound or severe disability living in the community | 3.360 | (1.298) | 3.532 | (1.077) |
| Proportion of people with high/very high level of psychological distress | 10.901 | (1.952) | 10.978 | (1.915) |
| Health risk factors (%) |  |  |  |  |
| Current smokers | 20.274 | (3.846) | 20.353 | (3.755) |
| Alcohol consumption at levels of high risk to health | 5.833 | (2.261) | 5.835 | (2.261) |
| Physical inactivity | 35.719 | (6.132) | 35.993 | (6.053) |
| Obese persons | 17.939 | (3.238) | 18.029 | (3.233) |
| Access to services (%) |  |  |  |  |
| Delayed purchasing prescribed medication | 8.683 | (2.781) | 8.668 | (2.748) |
| Have difficulty in accessing service | 25.127 | (4.586) | 25.034 | (4.544) |
| Have difficulty in transportation | 3.015 | (0.871) | 3.031 | (0.867) |
| Physician density |  |  |  |  |
| Number of specialists per 1,000 population | 0.950 | (2.188) | 0.850 | (2.589) |
| Number of GPs per 1,000 population | 1.034 | (0.537) | 1.095 | (0.503) |
| Number of EDs by SLAs |  |  |  |  |
| No EDs | 0.539 | (0.499) | 0.382 | (0.486) |
| 1-2 EDs | 0.347 | (0.476) | 0.440 | (0.497) |
| 3 or more EDs | 0.114 | (0.318) | 0.177 | (0.382) |
