# Supplementary material for: What explains the regional variation in the use of general practitioners in Australia?
Source: BMC Health Serv Res. 2020 Apr 19;20:325. doi: 10.1186/s12913-020-05137-1 (PMC7168818; doi:10.1186/s12913-020-05137-1)
Supplement: Supplementary file 2 — Additional file 2: Figure S1. Coefficients of variables across quantiles. [file 12913_2020_5137_MOESM2_ESM.docx]

Additional file 2


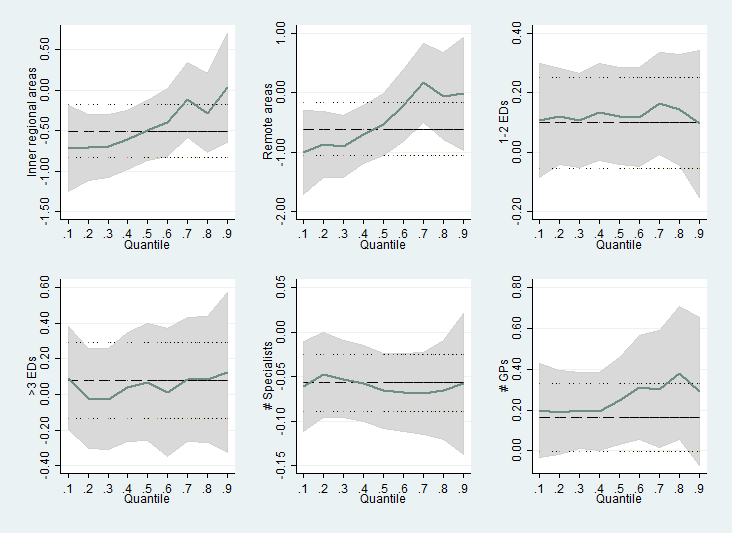


Figure A1: Coefficients of variables across quantiles

*Notes*: Each panel shows a specific quantile regression coefficient with the corresponding confidence interval. The coefficients from the OLS regression are represented by the horizontal black line. Only the coefficients for six explanatory variables that measure the capacity of the health care system and the accessibility of health care are plotted here and the rest of others are available upon request.
