# Supplementary material for: What explains the regional variation in the use of general practitioners in Australia?
Source: BMC Health Serv Res. 2020 Apr 19;20:325. doi: 10.1186/s12913-020-05137-1 (PMC7168818; doi:10.1186/s12913-020-05137-1)
Supplement: Supplementary file 3 — Additional file 3: Table S2. Estimation results for regional variation in GP use by rurality. [file 12913_2020_5137_MOESM3_ESM.docx]

Additional file 3

Table A2: Estimation results for regional variation in GP use by rurality

|  | Dependent variable: Number of GP visits per capita by SLAs | | | |
| --- | --- | --- | --- | --- |
| Variables | Major city and inner regional areas | | Rural and remote areas | |
| Age distribution (base is age 30-44, %) |  |  |  |  |
| Age 0-9 | -0.096*** | (0.030) | 0.061 | (0.068) |
| Age 10-29 | -0.046*** | (0.015) | 0.087* | (0.045) |
| Age 45-64 | -0.009 | (0.019) | -0.004 | (0.040) |
| Age 65 and above | 0.085** | (0.033) | 0.027 | (0.062) |
| Share of male | -0.039 | (0.034) | 0.052 | (0.049) |
| SEIFA-IRSD index (base is above 75th percentile - the most advantaged) |  |  |  |  |
| 25th percentile and below (the most disadvantaged) | -0.441* | (0.260) | -0.573 | (0.427) |
| 25th-50th percentile | -0.307 | (0.195) | -0.828** | (0.341) |
| 50th-75th percentile | -0.044 | (0.120) | -0.504* | (0.271) |
| Proportion of Aboriginal population | -0.009 | (0.042) | 0.016 | (0.020) |
| Proportion of concession card holders | 0.092*** | (0.015) | 0.020 | (0.019) |
| Share of fair or poor self-assessed health population | 0.002 | (0.037) | 0.007 | (0.040) |
| Chronic disease and conditions (%) |  |  |  |  |
| Type 2 diabetes | -0.686*** | (0.194) | 0.067 | (0.345) |
| Respiratory system disease | -0.015 | (0.014) | 0.026 | (0.046) |
| Circulatory system disease | -0.111*** | (0.020) | 0.115** | (0.047) |
| Proportion of people with profound or severe disability living in the community | 0.226** | (0.104) | 0.293** | (0.136) |
| Proportion of people with high/very high level of psychological distress | 0.116** | (0.055) | -0.180 | (0.134) |
| Health-related factors (%) |  |  |  |  |
| Current smokers | -0.098*** | (0.032) | -0.015 | (0.065) |
| Alcohol consumption at levels of high risk to health | -0.100* | (0.054) | 0.003 | (0.035) |
| Physical inactivity | 0.036** | (0.016) | 0.016 | (0.026) |
| Obese persons | 0.152*** | (0.030) | 0.010 | (0.054) |
| Access to services (%) |  |  |  |  |
| Delayed purchasing prescribed medication because could not afford it | 0.034 | (0.027) | 0.008 | (0.061) |
| Have difficulty in accessing service | -0.099*** | (0.016) | -0.028 | (0.046) |
| Have difficulty in transportation | -0.273*** | (0.078) | 0.079 | (0.201) |
| Physician density |  |  |  |  |
| Number of specialists per 1,000 population | -0.061*** | (0.015) | -0.267* | (0.152) |
| Number of GPs per 1,000 population | 0.341*** | (0.105) | 0.052 | (0.139) |
| Number of EDs by SLAs (base is no EDs) |  |  |  |  |
| 1-2 EDs | 0.072 | (0.071) | 0.005 | (0.245) |
| 3 or more EDs | -0.005 | (0.141) | 0.106 | (0.284) |
| Constant | 11.246*** | (2.012) | -3.868 | (5.174) |
| Number of observations | 541 | | 215 | |
| R squared | 0.612 | | 0.556 | |

*Notes:* Numbers in parentheses are white robust standard errors. Significant level * p<0.10, ** p<0.05, *** p<0.01.
