# Supplementary material for: What explains the regional variation in the use of general practitioners in Australia?
Source: BMC Health Serv Res. 2020 Apr 19;20:325. doi: 10.1186/s12913-020-05137-1 (PMC7168818; doi:10.1186/s12913-020-05137-1)
Supplement: Supplementary file 4 — Additional file 4: Figure S2. Unexplained differences in GP visits between the decile with lowest GP usage and higher-usage deciles. [file 12913_2020_5137_MOESM4_ESM.docx]

Additional file 4

Figure A2: Unexplained differences in GP visits between the decile with lowest GP usage and higher-usage deciles

*Notes:* The unexplained differences in GP visits per capita were expressed as coefficients of decile dummy variables. The division into ten deciles was based on unadjusted GP visits per capita. The three models were a series of linear multiple linear regression models that used GP visits per capita as the dependent variable and differed in the sets of covariates added as independent controls. The first model contained only dummy variables for the deciles, representing the differences in GP usage across the deciles. The second model added the variables from demand-side, such as age, gender, and health-related indicators. The third model contained supply-side factors as well as demand-side variables: the density of GP and specialists and the number of EDs. All the estimated coefficients were statistically significant.
